# In Vitro Fermentation of Polysaccharide from Edible Alga *Enteromorpha clathrata* by the Gut Microbiota of Patients with Ulcerative Colitis

**DOI:** 10.3390/nu15194122

**Published:** 2023-09-24

**Authors:** Mingfeng Ma, Min Quan, Jiaxue Zhang, Aijun Zhang, Puyue Gao, Qingsen Shang, Guangli Yu

**Affiliations:** 1 Key Laboratory of Marine Drugs of Ministry of Education, Shandong Provincial Key Laboratory of Glycoscience and Glycotechnology, School of Medicine and Pharmacy, Ocean University of China, Qingdao 266003, China; mmf621121@163.com (M.M.); q15265991920@163.com (M.Q.); zjx03120959@163.com (J.Z.); 2 Laboratory for Marine Drugs and Bioproducts, Qingdao National Laboratory for Marine Science and Technology, Qingdao 266003, China; 3 Qilu Hospital of Shandong University (Qingdao), Qingdao 266035, China; zajstj@163.com (A.Z.); gaopuyue@163.com (P.G.); 4 Qingdao Marine Biomedical Research Institute, Qingdao 266071, China

**Keywords:** gut microbiota, ulcerative colitis, marine polysaccharide, *E. clathrata*, chronic diseases, next-generation prebiotics, fermentation

## Abstract

Dietary intake of the sulfated polysaccharide from edible alga *E. clathrata* (ECP) has recently been illustrated to attenuate ulcerative colitis (UC) by targeting gut dysbiosis in mice. However, ECP is not easily absorbed in the gut and, as a potential candidate for next-generation prebiotics development, how it is fermented by human gut microbiota has not been characterized. Here, using in vitro anaerobic fermentation and 16S high-throughput sequencing, we illustrate for the first time the detailed fermentation characteristics of ECP by the gut microbiota of nine UC patients. Our results indicated that, compared to that of glucose, fermentation of ECP by human gut microbiota produced a higher amount of anti-inflammatory acetate and a lower amount of pro-inflammatory lactate. Additionally, ECP fermentation helped to shape a more balanced microbiota composition with increased species richness and diversity. Moreover, ECP significantly stimulated the growth of anti-colitis bacteria in the human gut, including *Bacteroides thetaiotaomicron*, *Bacteroides ovatus*, *Blautia* spp., *Bacteroides uniformis*, and *Parabacteroides* spp. Altogether, our study provides the first evidence for the prebiotic effect of ECP on human gut microbiota and sheds new light on the development of ECP as a novel prebiotic candidate for the prevention and potential treatment of UC.

## 1. Introduction

*E. clathrata* is an edible seaweed that has been traditionally consumed in different coastal areas of East China and other Asian countries [1,2,3]. Recently, dietary intake of the sulfate polysaccharide from *E. clathrata* (ECP) was observed to attenuate dextran sulfate sodium (DSS)-induced UC in mice [1]. Mechanistically, ECP ameliorated intestinal mucosal damage by significantly promoting the growth of the anti-colitis bacterium *P. distasonis* in the gut microbiota of DSS-fed mice [1]. Apart from ulcerative colitis, oral administration of ECP was also found to exert therapeutic effects on loperamide-induced constipation and high-fat-diet-induced obesity [3,4]. Similarly, the beneficial effects of ECP on constipation and obesity were also found to be correlated with the changes in gut microbiota [3,4]. Taken together, these studies suggest that ECP is a candidate next-generation prebiotic for the prevention and potential treatment of gut dysbiosis-associated chronic diseases [1,2,3,4].

As a complex marine natural polysaccharide, ECP is not easily absorbed in the gastrointestinal tract after oral administration due to its polyanionic nature and large molecular weight [5,6]. Accumulating evidence has indicated that ECP could be readily fermented and utilized by specific anaerobes in the mouse gut, including *E. xylanophilum*, *Parabacteroides* spp., *Bifidobacterium* spp., *A. muciniphila*, and *Lactobacillus* spp. [1,2,3,4]. Fermentation of ECP by the mouse gut microbiota stimulated the growth of beneficial short-chain fatty acid (SCFA)-producing bacteria and changed the metabolic functions of the intestinal microbiome [1,2,3,4]. However, although the interactions between ECP and the murine gut microbiota have been extensively studied, how it is fermented by the human intestinal microbiota has not been investigated.

The human gastrointestinal tract is home to tens of trillions of different gut anaerobes [5,6]. Dissecting the complex interactions between the human gut microbiota and dietary polysaccharides would aid in the development of next-generation prebiotics from natural resources [5,6]. In the present study, using an in vitro anaerobic fermentation system and combined with 16S rRNA high-throughput sequencing, we demonstrate for the first time the detailed fermentation characteristics of ECP by the gut microbiota of nine patients with UC. Our study provides the first evidence for the prebiotic effect of ECP on human gut microbiota and sheds new light on the development of ECP as a novel prebiotic candidate for the prevention and potential treatment of UC and other chronic diseases by targeting gut dysbiosis.

## 2. Materials and Methods

### 2.1. Chemicals and Reagents

ECP was used and prepared as previously described [1,2,3]. ECP was mainly composed of rhamnose (49.7%), glucose (29.9%), glucuronic acid (10.8%), and xylose (7.2%). In addition, small amounts of mannose (1.0%) and galactose (1.3%) were also detected. The molecular weight of ECP was 11.67 kDa, and the sulfate content of ECP was 14.7%. Bacterial nutrients used for the in vitro anaerobic fermentation were all obtained from Sigma-Aldrich (St. Louis, MO, USA). The standard SCFA solutions, including lactate, acetate, propionate, and butyrate, were also obtained from Sigma-Aldrich (St. Louis, MO, USA). Hemin and L-cysteine hydrochloride used for the fermentation were purchased from Sangon Biotech (Shanghai, China). All other analytical-grade chemicals were acquired from Sinopharm Chemical (Shanghai, China).

### 2.2. In Vitro Anaerobic Fermentation of ECP by the Human Gut Microbiota

Nine patients diagnosed with UC were recruited for the research. The fresh fecal samples from the UC patients were anaerobically collected using the method previously described [7]. The well-established VI culture medium was applied to explore the in vitro fermentation characteristics of ECP by the gut microbiota of UC patients [1,7]. The simple sugar glucose was used as a control for the in vitro fermentation experiments. The complex carbohydrate ECP or the simple sugar glucose was added to the VI medium as a major carbon source at a concentration of 8 g/L. The fermentation experiment was carried out at 37 °C in an Electrotek AW 500SG anaerobic (80% N_2_, 10% H_2_, and 10% CO_2_) chamber (Shipley, UK).

### 2.3. Carbohydrate Utilization and SCFA Analyses

After 48 h, the fermentation experiment was terminated, and the obtained media were collected for carbohydrate utilization analysis and SCFA analysis. The phenol–sulfuric acid (PSA) method was applied to investigate the utilization of glucose and ECP during fermentation [1,7]. The SCFA concentrations in the fermentation media were determined and analyzed using a high-performance liquid chromatograph (HPLC) (Agilent 1260, Santa Clara, CA, USA) equipped with an Aminex HPX-87H column (Bio-Rad, Hercules, CA, USA) [1,7].

### 2.4. High-Throughput Sequencing and Bioinformatic Analyses

The metagenomic DNAs of the different human gut microbiota were extracted from the fecal samples and the fermentation media using Qiagen QIAamp DNA Stool Mini Kit (Hilden, Germany). The 16S hypervariable gene regions (V3 to V4) were specifically amplified using the two well-established universal primers 338F and 806R [1,7]. The 16S amplicons were quality checked and sequenced using Illumina PE300 platform from the Majorbio Bio-pharm Biotechnology (Shanghai, China). The bioinformatic analyses of the gut microbiota, including heatmap analysis, alpha diversity analysis, and beta diversity analysis, were all conducted using the online tools from the Majorbio Cloud Platform (www.majorbio.com (accessed on 25 June 2023)). The PICRUSt2 algorithm was applied to investigate the metabolic functions of the human gut microbiota, as previously described [8].

### 2.5. Statistical Analyses

All results were expressed as the mean ± standard error of mean (SEM). The statistical analyses were performed using Student’s t-test from the GraphPad Prism 8.0.2 software (San Diego, CA, USA). The Wilcoxon rank-sum test was performed to compare the structural differences of the gut microbiota between two groups. The results were considered statistically significant at *p* < 0.05.

## 3. Results

### 3.1. ECP Was Utilized and Fermented to Produce SCFAs by the Gut Microbiota of UC Patients

**Figure 1:**
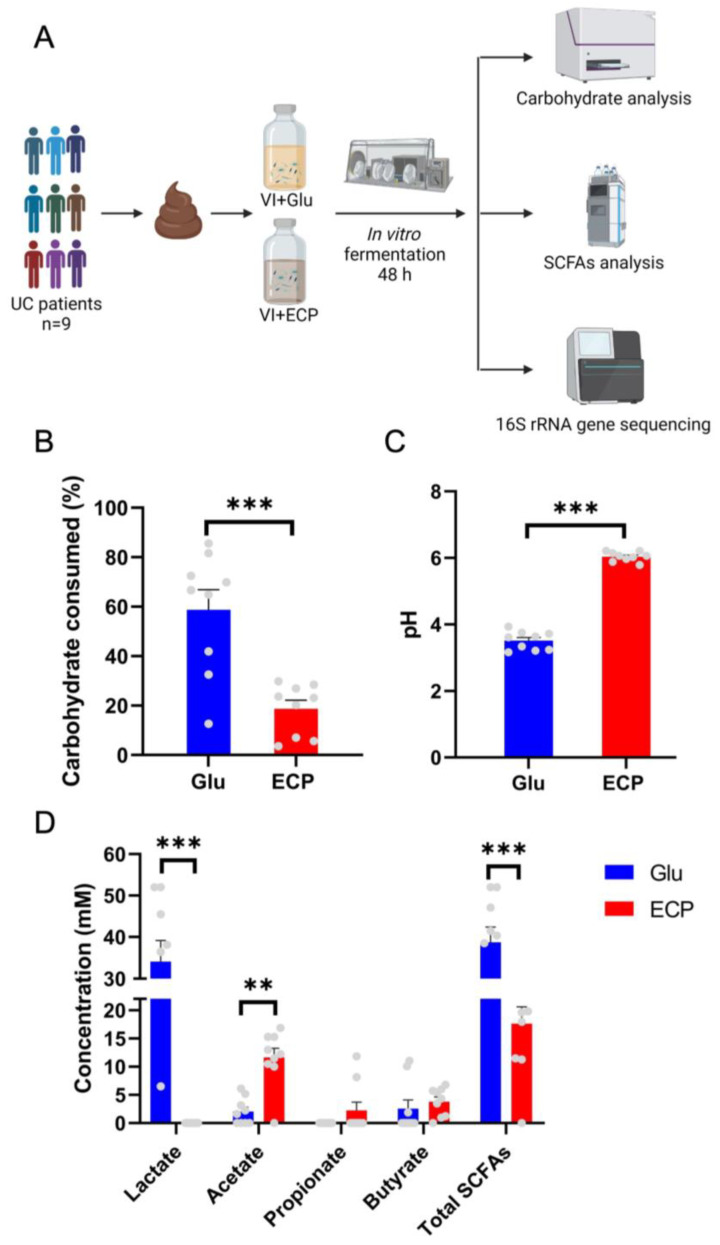
Fermentation of ECP by the gut microbiota of UC patients. Experimental design (**A**). A total of 9 UC patients were recruited for the present study. The fecal samples were collected anaerobically. Carbohydrate utilization analysis (**B**). pH of the fermentation medium (**C**). Production of the SCFAs, including lactate, acetate, propionate, and butyrate (**D**). ** *p* < 0.01; *** *p* < 0.001. Part of the figure was created with BioRender.com (accessed on 25 June 2023).

Preceding studies have indicated that ECP is a good prebiotic candidate for the prevention and treatment of UC from marine resources [1,9,10]. Dietary intake of ECP significantly improved the UC symptoms and ameliorated the intestinal mucosal damage in DSS-fed mice [1]. However, as a complex polysaccharide with a high molecular weight, ECP is not easily absorbed in the gastrointestinal tract after oral administration, and how it is fermented and utilized by the human gut microbiota has not been characterized [5,6]. Here, using an in vitro anaerobic fermentation system, we found that ECP could be utilized and fermented to produce SCFAs by the gut microbiota of patients with UC (Figure 1A–D). However, the human gut microbiota could only steadily degrade and ferment about 20% of the complex carbohydrate ECP (Figure 1B,C). In contrast, the human gut microbiota fermented about 60% of the simple sugar glucose during the same fermentation period (Figure 1B).

Gut microbiota fermentation of polysaccharides would produce significant amounts of SCFAs and, thus, decrease the pH in the medium [1,5]. In the present study, we observed that fermentation of the simple sugar glucose resulted in a more dramatic decrease in the pH as compared to that of ECP (Figure 1C). As for the production of SCFAs, it is of interest to find that glucose fermentation produced a higher amount of lactate, while ECP fermentation produced more acetate (Figure 1D). Apart from lactate and acetate, small amounts of propionate and butyrate were also produced during fermentation. The divergent fermentation characteristics of ECP and glucose indicate that these two carbohydrates were utilized and fermented by different anaerobes in the human gut.

### 3.2. ECP Fermentation Helped to Shape A More Balanced Composition of the Human Gut Microbiota with Increased Species Richness and Diversity

**Figure 2:**
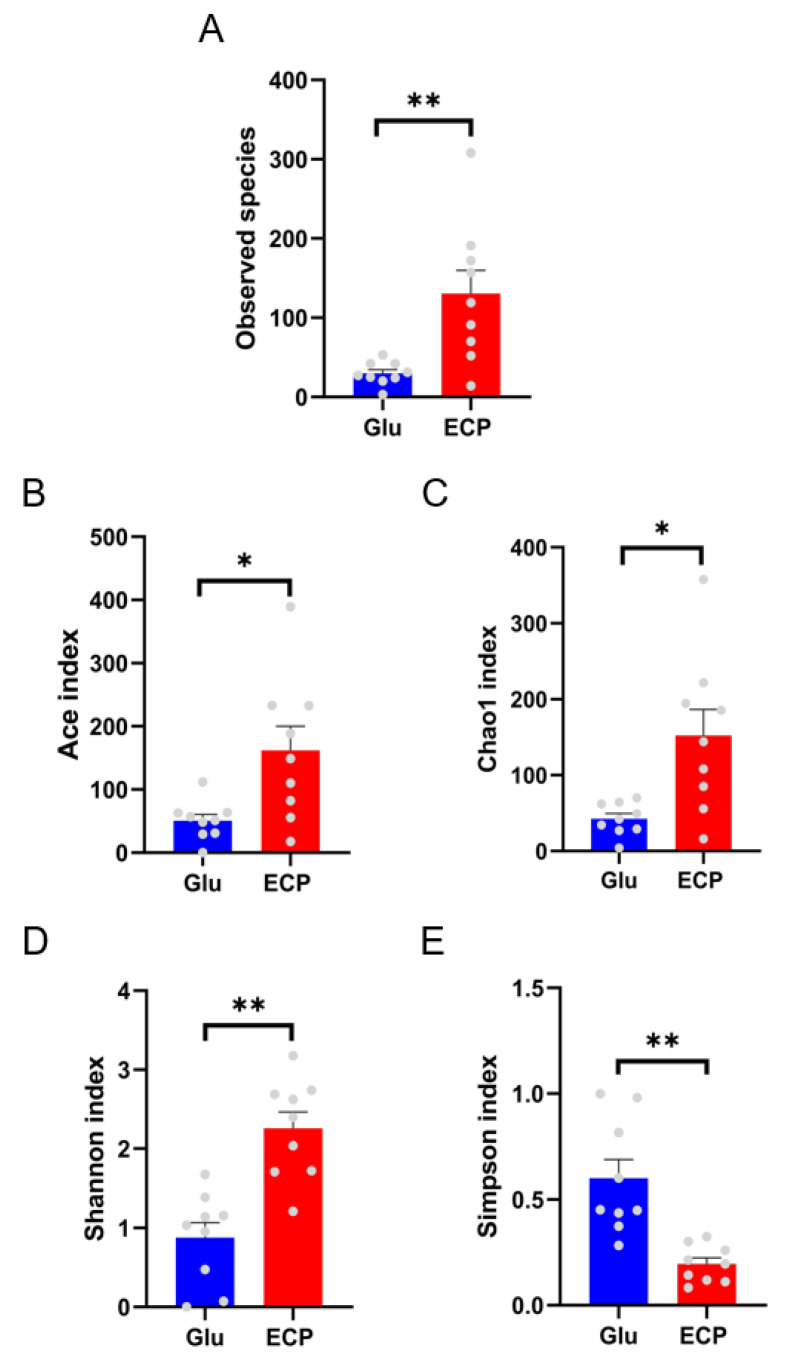
Alpha−diversity analysis of the 16S sequencing data of the human gut microbiota. Observed species (**A**). The number of the observed species was analyzed to investigate how many species were presented after fermentation. It is a marker of species richness. Abundance-based coverage estimators (ACE) index (**B**). Chao1 index (**C**). Shannon index (**D**). Simpson index (**E**). These are common alpha diversity indices. * *p* < 0.05; ** *p* < 0.01.

Further, 16S rRNA high-throughput sequencing and bioinformatic analyses were utilized to further explore the fermentation characteristics of ECP by the human gut microbiota. Alpha diversity analysis of the human gut microbiota suggested that the fermentation of ECP helped to shape a more balanced composition of the human gut microbiota with increased species richness and diversity (Figure 2A–E). This indicates that more species of bacteria were needed for the degradation and utilization of the complex carbohydrate ECP as compared to that of simple sugar glucose during the fermentation processes.

**Figure 3:**
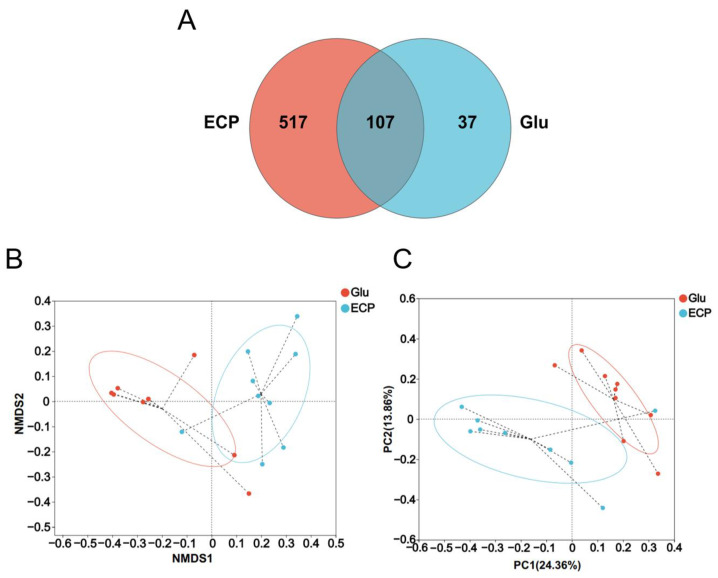
Beta diversity analysis of the 16S sequencing data of the human gut microbiota. Venn diagram analysis based on the operational taxonomic units (**A**). The ECP group and the Glu group share 107 OTUs. However, 517 OTUs were only found in the ECP group and 37 OTUs were only found in the Glu group. Non-metric multidimensional scaling (NMDS) score plot analysis (**B**). Principal components analysis (PCA) score plot analysis (**C**). A clear separation of the structure of the two gut microbial communities was observed in both NMDS score plot analysis and PCA score plot analysis. This indicates that the composition of the gut microbiota in the ECP treatment group and the glucose treatment group was quite different.

Beta diversity analysis of the 16S sequencing data further confirmed that ECP and glucose modulated the human gut microbiota towards different communities during the fermentation processes (Figure 3A–C). This indicates that the fermentation of glucose and ECP was driven by different functional microbes from the human gut microbiota.

### 3.3. ECP Promoted the Growth of Anti-Colitis Bacteria, Including B. thetaiotaomicron, B. ovatus, B. uniformis, and Parabacteroides *spp.*, during Fermentation

**Figure 4:**
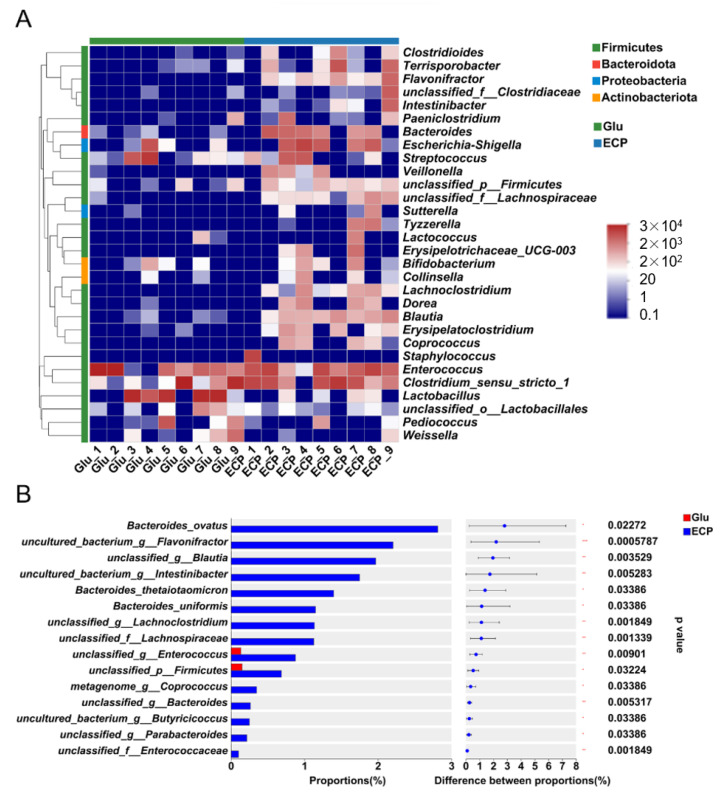
Compositional analysis of the human gut microbiota. Heatmap analysis of the gut microbiota at the genus level (**A**). Only the top 30 most abundant genera were shown in the figure. The genera belonging to different phylums were also identified. Wilcoxon rank-sum test analysis of the gut microbiota at the species level (**B**). Different species of the human gut bacteria were ranked by their proportions in the medium. * *p* < 0.05; ** *p* < 0.01; *** *p* < 0.001.

To identify the keystone bacteria that drive the fermentation of ECP, we performed the heatmap and Wilcoxon rank-sum test (Figure 4 and Figure S1) analyses. At the phylum level, the human gut microbiota were dominated by *Firmicutes*, *Bacteroidota*, *Protaobactoria*, and *Actinobacteriota* (Figure 4A). Interestingly, we found that at the genus level, the probiotic acetate-producing bacteria, including *Blautia* spp. and *Parabacteroides* spp., were more abundant in the ECP treatment group than that in the glucose treatment group (Figure 4A and Figure S1). It is highly possible that ECP was degraded and utilized by *Blautia* spp. and *Parabacteroides* spp. to produce acetate during fermentation. This could help to explain why more acetate was produced during ECP fermentation (Figure 1D). Additionally, it should be noted that our previous studies demonstrated that ECP could be fermented by the human gut anaerobe *P. distasonis* F1-28 to produce acetate [1]. Collectively, these results provide further evidence for the utilization of ECP by specific microbes in the human gut.

**Figure 5:**
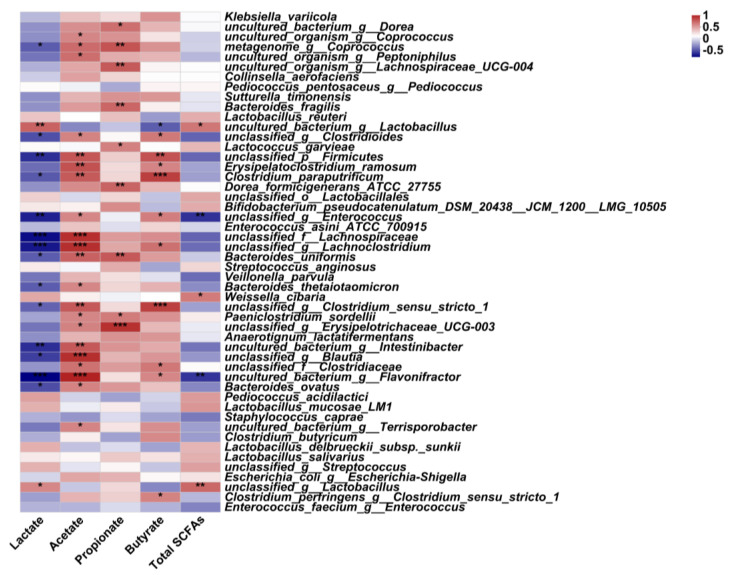
Spearman’s correlation analysis of the human gut microbiota with productions of the lactate, acetate, propionate, butyrate and total SCFAs. Positive correlations were marked in red and negative correlations were marked in blue. The analysis was conducted at the species level. Correlations with R > 0.3 or R < −0.3 were identified by asterisks. * *p* < 0.05; ** *p* < 0.01; *** *p* < 0.001.

At the species level, it is of interest to note that, as compared to that of glucose, ECP significantly promoted the growth of anti-colitis bacteria, including *B. thetaiotaomicron*, *B. ovatus*, and *B. uniformis*, during the fermentation process (Figure 4B). Moreover, the production of acetate, a major fermentation product of ECP, was also found to be positively correlated with the abundances of *B. thetaiotaomicron*, *B. ovatus*, and *B. uniformis* (Figure 5). Collectively, these results suggest that ECP could potentially be used as a next-generation prebiotic for the prevention and potential treatment of gut dysbiosis in UC patients by stimulating the growth of probiotic bacteria, including *B. thetaiotaomicron*, *B. ovatus*, and *B. uniformis*.

### 3.4. ECP Changed the Metabolic Functions of the Human Gut Microbiota

**Figure 6:**
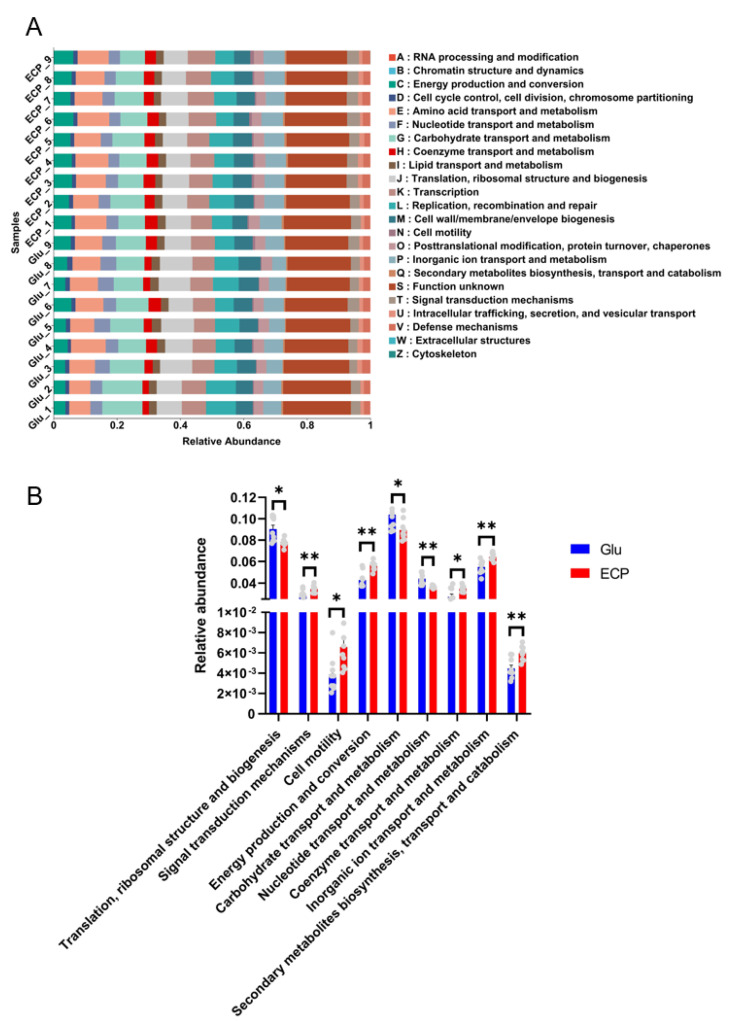
Metabolic function analysis of the human gut microbiota using the PICRUSt2 algorithm. COG function classification (**A**). Different metabolic functions were predicted and analyzed. Comparison of the metabolic functions between the two groups (**B**). * *p* < 0.05; ** *p* < 0.01.

The PICRUSt2 algorithm has been widely used for the prediction of metagenome functions of the human gut microbiota [9]. We next asked what effects ECP and glucose have on the metabolism of the human gut microbiota during fermentation. Clusters of orthologous gene (COG) function analysis based on the 16S sequencing data indicated that adding ECP to the culture medium significantly changed the metabolic functions of the human gut microbiota, including translation, ribosomal structure and biogenesis (TRSB), signal transduction mechanisms (STM), cell motility (CM), energy production and conversion (EPC), carbohydrate transport and metabolism (CarTM), nucleotide transport and metabolism (NTM), coenzyme transport and metabolism (CoTM), inorganic ion transport and metabolism (IITM), and secondary metabolite biosynthesis, transport, and catabolism (SMBTC) (Figure 6A,B). Specifically, compared to that of simple sugar glucose, ECP up-regulated the metabolic functions of EPC and SMBTC while down-regulating the metabolic function of CarTM (Figure 6B). Collectively, these results suggest that ECP and glucose could exert different effects on the metabolism of the human gut microbiota.

## 4. Discussion

The oral administration of ECP has recently been proposed as a potential new therapeutic approach for the treatment of gut dysbiosis-associated diseases, including UC, obesity, and chronic constipation [1,2,3,4,8,10]. Nonetheless, as a marine natural polysaccharide, ECP is not easily absorbed in the gastrointestinal tract after oral administration due to its polyanionic nature and large molecular weight [5,6]. Therefore, when reaching the cecum and distal colon, it could be fermented and utilized by specific microbes in the human gut [5,11,12]. Previous animal studies have indicated that *A. muciniphila*, *Parabacteroides* spp., *Bifidobacterium* spp., *Lactobacillus* spp., and *E. xylanophilum* are candidate fermenters of ECP in the mouse gut [1,2,3,4]. However, in humans or UC patients, how ECP is fermented and metabolized by the gut microbiota has not been investigated. Here, using in vitro anaerobic fermentation and 16S rRNA high-throughput sequencing, we illustrate for the first time the detailed fermentation characteristics of ECP by the gut microbiota of nine patients with UC. Our study provides the first evidence for the prebiotic effect of ECP on the human gut microbiota and sheds new light on the development of ECP as a novel prebiotic candidate for the prevention and potential treatment of UC and other chronic diseases by targeting gut dysbiosis.

Accumulating studies have indicated that the low fecal pH and high lactate levels are causally related to the development of UC in humans [13,14,15,16,17]. In the present study, we found that fermentation of the simple sugar glucose by the gut microbiota of UC patients produced a significantly higher amount of lactate and resulted in a more dramatic decrease in pH as compared to that of ECP. As a complex carbohydrate, ECP was slowly fermented by the human gut microbiota and, as a consequence, we only observed a mild decrease in the pH. Moreover, ECP fermentation produced no lactate but a high proportion of acetate. In contrast to lactate, which, in some cases, is a pro-inflammatory molecule in the pathogenesis of UC [15,16,17,18], acetate has been illustrated to have potent anti-inflammatory effects [19,20,21]. By applying the organoid-derived epithelial monolayer cultures from UC patients, previous studies have well illustrated that a high acetate concentration in the colon could help to protect the integrity of the intestinal barrier [19]. In addition, the anti-inflammatory drug evodiamine was also found to exert its therapeutic effects on UC by increasing the colonic concentration of acetate [22]. Collectively, these results indicated that ECP could possibly help to increase the colonic acetate concentration in UC patients. Our study suggests that ECP is a good source of dietary fiber for the prevention and potential treatment of inflammatory bowel diseases, including UC and Crohn’s disease.

Carbohydrate-active enzymes (CAZymes), including glycoside hydrolases (GHs), glycosyltransferases (GTs), polysaccharide lyases (PLs), carbohydrate esterases (CEs), carbohydrate-binding modules (CBMs), and auxiliary activities (AAs), are the most important catalytic enzymes for the metabolism of complex dietary polysaccharides in the human gut [5,23,24,25,26]. *Bacteroides* spp. is armed with a plethora of finely tuned CAZymes and has been proposed as a keystone genus for the degradation of complex natural polysaccharides in the human colon [23,24,25,26]. For example, *Bacteroides xylanisolvens* AY11-1, a probiotic anaerobe isolated from the healthy human colon, could degrade and ferment alginate in our daily diet [7]. Fermentation of alginate and its derivatives by *B. xylanisolvens* AY11-1 produced remarkable amounts of oligosaccharides and SCFAs [7]. Further, *B. thetaiotaomicron*, *B. ovatus*, and *B. uniformis* have also been demonstrated to degrade and ferment chondroitin sulfate, laminarin, porphyrin, and agar [5]. Our results indicated that *B. thetaiotaomicron*, *B. ovatus*, and *B. uniformis* are three major fermenters of ECP from the human gut. Interestingly, *B. thetaiotaomicron*, *B. ovatus*, and *B. uniformis* have all been identified to have potent anti-colitis effects in UC mice [27,28,29,30,31]. Oral intake of these three probiotic bacteria could remodel the immune microenvironment in the colon and reduce the inflammatory response caused by DSS and trinitrobenzene sulfonic acid (TNBS) [27,28,29,30,31]. As compared to that of glucose, ECP significantly stimulated the growth of the three probiotic bacteria in the gut microbiota of UC patients. Our study suggests that ECP might be used as a potential novel prebiotic agent for the prevention and potential treatment of gut dysbiosis in UC patients. However, more detailed clinical and animal studies are needed to test this possibility.

*Blautia* spp. and *Parabacteroides* spp. are two genera of acetate-producing next-generation probiotic bacteria that were highly enriched in the medium during ECP fermentation [1,32,33,34,35,36]. Similar to that of the *Bacteroides* spp., *Blautia* spp. and *Parabacteroides* spp. have also been demonstrated to have robust anti-inflammatory effects in both DSS-induced colitis and TNBS-induced colitis [1,32,33,34,35,36]. Specifically, *B. producta* could ameliorate DSS-induced UC by inhibiting the toll-like receptor 4/nuclear factor-kappa B pathway, suppressing the inflammatory responses in the colon, and modulating the composition of the dysbiotic gut microbiome [32]. In addition, specific strains of *P. distasonis* could prime the dendritic cells to induce regulatory T cells from naïve CD4+ T cells [36]. Moreover, our previous study indicated that ECP could promote the growth of *P. distasonis* F1-28, a next-generation anti-colitis probiotic bacterium isolated from the health human colon, and increase its production of beneficial SCFAs [1]. Apart from *P. distasonis*, it is highly possible that other species of bacteria are also capable of fermenting and utilizing ECP within the genera of *Blautia* spp. and *Parabacteroides* spp. Nevertheless, future studies are encouraged to further explore this issue.

The fermentation of marine polysaccharides by the human gut microbiota would produce a significant amount of SCFAs [1,5,7]. In line with the differences in the production of SCFAs, adding ECP to the culture medium remarkably changed the metabolic functions of the human gut microbiota. Specifically, ECP down-regulated the metabolic function of CarTM. This could help to explain why more complex carbohydrate ECP was left in the culture medium after fermentation as compared to that of simple sugar glucose. However, it should be noted that ECP also significantly up-regulated the metabolic functions of EPC and SMBTC. This indicates that although ECP fermentation produced less total SCFAs, it might have changed the production of secondary metabolites by the human gut microbiota. More detailed studies using metabolomics are, therefore, needed to further explore this issue.

Our study has some limitations. First, the sample size was not very big since we only had nine UC patients in the study. The composition of the gut microbiota varied between different UC patients [37,38,39], and more detailed studies with a larger sample size are needed to further explore the prebiotic effect of ECP on human gut microbiota. Second, due to the lack of enough fecal samples, we did not check the initial gut microbiota composition before fermentation. We only focused on the different effects of glucose and ECP on the gut microbiota during fermentation in the present study. Although many studies have indicated that UC patients are characterized with a dysbiotic gut microbiome [37,39], how this would affect the fermentation outcomes of ECP and other dietary polysaccharides has not been fully determined. Therefore, more studies with healthy controls are urgently needed. Third, as a complex dietary fiber, although theoretically ECP is not easily absorbed and digested in the upper-digestion tract, we did not conduct relevant simulated digestion experiments to check the bio-accessibility of the ECP to the gut microbiota in the present study. Part of the reason is that we currently do not have a good analytical method to study the metabolism and biotransformation of ECP in the gut after oral administration. More studies are still needed to fully characterize the in vitro and in vivo metabolic fates of ECP in the human gastrointestinal tract since this could affect the bioactivities of the algal extracts [40].

## 5. Conclusions

Using in vitro anaerobic fermentation and 16S rRNA high-throughput sequencing, we illustrate for the first time the detailed fermentation characteristics of ECP by the gut microbiota of nine patients with UC. Our results indicated that compared to that of simple sugar glucose, the fermentation of complex carbohydrate ECP by the human gut microbiota produced a higher amount of anti-inflammatory acetate and a lower amount of pro-inflammatory lactate. Additionally, ECP fermentation helped to shape a more balanced composition of the human gut microbiota, with increased species richness and diversity. Moreover, ECP significantly promoted the growth of anti-colitis bacteria in the human gut, including *B. thetaiotaomicron*, *B. ovatus*, *B. uniformis*, *Blautia* spp., and *Parabacteroides* spp. Altogether, our study provides the first evidence for the prebiotic effect of ECP on human gut microbiota and sheds new light on the development of ECP as a novel prebiotic candidate for the prevention and potential treatment of UC and other chronic diseases by targeting gut dysbiosis.

## Data Availability

The data are available on request from the corresponding authors.

## Supplementary Materials

The following supporting information can be downloaded at: https://www.mdpi.com/article/10.3390/nu15194122/s1, Figure S1: Wilcoxon rank-sum test analysis of the gut microbiota at the genus level.
