# Supplementary figures and images for: In Vitro Fermentation of Polysaccharide from Edible Alga Enteromorpha clathrata by the Gut Microbiota of Patients with Ulcerative Colitis
Source: Nutrients. 2023 Sep 24;15(19):4122. doi: 10.3390/nu15194122 (PMC10574352; doi:10.3390/nu15194122)

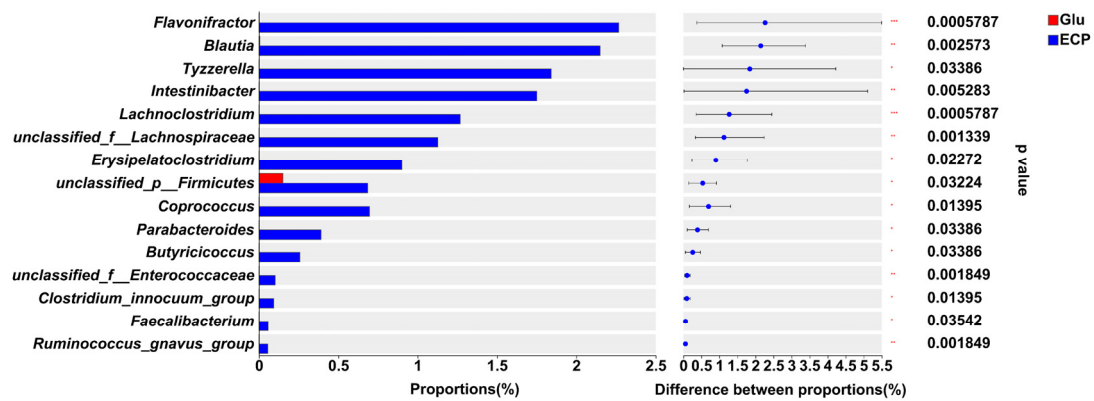

Figure S1. Wilcoxon rank-sum test analysis of the gut microbiota at the genus level.

Supplement: Supplementary file 1 [file nutrients-15-04122-s001.zip › nutrients-2608579-supplementary.pdf]
